# Prevalence of genotypes and subtypes of hepatitis B viruses in Bangladeshi population

**DOI:** 10.1186/s40064-016-1840-2

**Published:** 2016-03-05

**Authors:** Md. Arifur Rahman, Farzana Hakim, Mamun Ahmed, Chowdhury Rafiqul Ahsan, Jamalun Nessa, Mahmuda Yasmin

**Affiliations:** Department of Microbiology, University of Dhaka, Dhaka, 1000 Bangladesh; Department of Biochemistry and Molecular Biology, University of Dhaka, Dhaka, Bangladesh; Department of Microbiology, Noakhali Science and Technology University, Sonapur, Noakhali, 3814 Bangladesh

**Keywords:** Genotype, Subtype, Mutation, HBsAg

## Abstract

The genetic variability of hepatitis B virus (HBV) represents a challenge for the sensitivity of immunologic and molecular based assays. Based on sequence divergences in the entire genome of >8 %, HBV genomes have been classified into ten genotypes designated as A to J. The aim of this study was to determine HBV genotypes and subtype in samples of HBV infected patients in Bangladesh. The sera samples were collected from chronically infected HBV patients. At first the DNA positive HBV samples were screened by EIA in our laboratory and the 1063 bp region of surface gene was amplified, sequenced and genotyped by sequence analysis. The same sequences were also used for subtypes and mutational analyses. After that, genotyping was also carried out by nested PCR using genotype specific primers in the same region of HBV surface gene. A total of 39 samples were sequencing to find out the genotypes and subtypes. It was found that the prevalent genotype was genotype C (subgenotype C1) which accounted for 48.7 %. The other genotypes found were genotype A (23.1 %) and genotype D (28.2 %). Predominant subtypes in Bangladesh were *adr* (41 %) followed by subtype *adw*2 (28.2 %), *ayw*3 (25.6 %), and others. Additionally, genotyping was also done by nested PCR using type-specific primers. In this method, out of 17 samples 6 were found to be genotype C, followed by genotype D (4 of 17) and genotype A (3 of 17). In PCR-based genotyping system we also observed the mix genotypes; 3 samples contained both genotype A and D, and 2 samples contained both C and D. The genetic diversity of HBV and distribution of its genotypes and subtypes amongst Bangladeshi population were done in this study, which will help us to provide information regarding circulating genotypes in this region and also help physicians to prescribe proper antiviral/interferon therapy.

**Electronic supplementary material:**

The online version of this article (doi:10.1186/s40064-016-1840-2) contains supplementary material, which is available to authorized users.

## Background

Hepatitis B virus (HBV) infection is one of the most widespread viral infections in humans with a spectrum of liver diseases ranging from asymptomatic state to serious public health problem (Lee 1997). About 2 billion people have been infected worldwide with an estimated 300–350 million carrier, 40 % of whom may develop complicated clinical sequelae, including liver cirrhosis (LC), liver cancer, and hepatocellular carcinoma (HCC) (Yang et al. 2008). According to WHO, approximately 600,000 deaths occur worldwide annually due to chronic complication of HBV associated liver disease (WHO 2008). The outcome of HBV infection depends on a wide variety of factors, such as, host’s immune response, rate of virus replication and probably genetic variability of the virus (Huy et al. 2003).

Prior to definition of genotypes, HBV were categorized as subtypes based on serological differences (Norder et al. 1992), based on immune reaction by amino acid pattern at specific location of “a” determinant which leads to the nine different subtypes designating *adw*2, *adw*4, *adr*q+, *adr*q−, *ayw*1, *ayw*2, *ayw*3, *ayw*4 and *ayr*. In 2002, a new subtype *adw*3 has been described (Norder et al. 1992; Magnius and Norder 1995). Their distributions within the genomic groups have shown to be restricted. Subtype *adw*2 is found to genotype A, B and C whereas; *adw*4 is only circulated on genotypes E and F (Norder et al. 1992). On the other hand, *ayw*2 and *ayw*3 both are associated with group D. However, *ayw*1 has been isolated from genotypes A and B (Norder et al. 1992). The HBV isolates expressing *adr*/*ayr* is only been found in genotype C (Norder et al. 1992).

Genotypically, HBV genomes have been classified according to their genetic variability (>8 % for whole genome) into ten major genotypes designating A to J (Magnius and Norder 1995; Cao 2009; Yu et al. 2010; Tatematsu et al. 2009). The genetic diversity occurs due to the replication error of the virus during their multiplication. Though HBV contains DNA molecules as their genetic material, it replicates via an RNA intermediate. That is why error rate of this virus is higher (approximately >2 × 10^4^ base substitutions/site/year) as the proofreading activity of the viral polymerase is lacking (Buti et al. 2005).

HBV genotypes show a distinct geographic and ethnic distribution. Genotype A is the most commonly distributed globally and is leading genotype in Europe, North America, Africa and India, whereas B and C are the dominant genotypes in East and Southeast Asia (Norder et al. 1993; Kao and Chen 2006). Genotype D is prevailing in the Middle East and Mediterranean region and India, and genotype E is commonly found in sub-Saharan Africa (Mulders et al. 2004; Kramvis et al. 2005) along with some other continents (Singh et al. 2009). Outside the Central and South America, genotypes F and H are rarely found (Alvarado Mora et al. 2011; Devesa et al. 2008), and Genotype G is circulating among the people of USA, Mexico, France and Germany (Tanwar and Dusheiko 2012). This genotype is normally present as a co-infection with other HBV genotypes, most commonly with genotype A. The genotypes I has been detected in Laos, Vietnam and China (Phung et al. 2010; Olinger et al. 2008), while the newest genotype; J was identified in the Ryukyu Islands in Japan (Tatematsu et al. 2009). The most commonly found genotypes in Asia are B and C with the exception of India where the genotype A and D are most prevalent (Cao 2009; Schaefer 2007; Acharya et al. 2006).

In this study, we attempted to find out the prevalence of HBV genotypes, subgenotypes and subtypes among the Bangladeshi population. The study also focused on clarifying the origin of these genotypes as well as some significant mutation on the S protein gene of HBV. Nucleotide substitution of these sites (mainly small S portion) may lead to changed amino acid sequences causing dramatic changes in viral life cycle.

## Methods

### Patients

Sera were collected from 50 cases chronically infected by HBV who attended one of the largest hospitals in the Dhaka city which provide services to the patients coming from different parts of the country. This hospital is well known for its diagnostic services and is therefore chosen by many physicians and patients. The samples were collected between the year 2009 and 2011, and were tested previously in the hospital laboratory by commercial real-time PCR (COBAS TaqManTM 48 Analyzer, Roche Diagnostics, Mannheim, Germany) to determine the HBV DNA level. The patients who cleared HBsAg within 6 months of onset of infection were excluded from this study as per physicians’ suggestion (Due to the possibility of acute infection). The age of the patients included in this study were 11–65 years with a mean age of 29.8 years. Patients’ sera were collected and stored at −20 °C until the experiment was done. After that, all sera samples were tested for HBsAg by enzyme immune assay (EIA) using commercial EIA kit (ETI-MAK-4, DiaSorin, Italy). The study was approved by Institutional ethical committee and all subjects gave their consent to participate in this study.

### DNA extraction and PCR amplification

HBV DNA was extracted from 200 µl of all sera samples regardless of EIA result, using a QIAamp DNA blood mini kit (Qiagen, Chatswort, Calif Germany) according to the manufacture’s instruction. A length of 1063 bp surface gene spanning from nt 2823–704 was amplified using primer pairs P1 (5′-TCA CCA TAT TCT TGG GAA CAA GA-3′) and S1-2 (5′-CGA ACC ACT GAA CAA ATG GC-3′). The reaction was carried out in 50 µl reaction volumes containing 200 pmol of each primer, 2.5 mM of each of the four dNTPs, 2.5 mM MgCl_2_, 1.25U of *Taq* DNA polymerase and 1 × PCR buffer. The thermocycler was programmed as 95 °C for 10 min, followed by 94 °C for 20 s, 55 °C for 20 s, 72 °C for 1 min and an additional 72 °C for 7 min. The resulting PCR product was detected by gel electrophoresis on a 0.8 % agarose gel. Genotype of HBV was determined by sequencing of amplified DNA and also by using genotype-specific PCR amplification.

### Genotyping using type specific primers

This is a rapid genotyping system proposed by Naito et al. (2001). The 1063 bp product generated by amplification with P1and S1-2 primers was used as template in this reaction. To determine the genotype (A to F), eight additional type specific primers were used. The same method was followed as well as same primers (Additional file 1) were used as proposed by Naito et al. in 2001. Here, two reaction mixtures were prepared, separated as mix A (for genotype A, B and C) and mix B (for genotype D, E and F). The reaction condition was as same as the first round reaction with a few changes in case of annealing temperature of primers. Genotypes were determined by their distinct pattern of band on agarose gel as genotype A (68 bp), B (281 bp), C (122 bp) for the mix A and D (119 bp), E (167 bp), and F (97 bp).

### Genotype determination by direct DNA sequence

The PCR products were purified using Microcon (Millipore) PCR purification kit according to the manufacture’s procedure, and the concentration of DNA was measured by nano-drop spectrophotometer. Each sample was sequenced by both forward and reverse primers. After ethanol precipitation, the purified PCR products were subjected to sequencing in the ABI prism 3130 Genetic analyzer (ABI prism, USA).

### Sequence analysis

Sequences received from ABI prism analyzer were ‘abi’ file format. The sequences were edited with Chromas 3.3 software (Tecnhnelysium Pty Ltd) by comparing with same DNA fragment of published sequences on GenBank and were saved as ‘scf’ file format. The contigs were assembled by DNASTAR’s Lasergene sequence analysis software (Burland 2000). The edited sequences were saved as FASTA file format for further analysis. A phylogenetic analysis was done using reference sequences from various countries retrieved from the international DNA data base (Additional file 2). Phylogenetic analysis of 950 bp fragment of large S gene using MEGA, version 5 (Tamura et al. 2007), was the basis for HBV genotyping implying p-distance substitution model with the neighbor joining method. The reliability of different phylogenetic groupings was evaluated using the bootstrap test (1000 bootstrap replications).

### Genotyping by NCBI genotyping tools

It is easy and rapid method and can be achieved using BLAST (basic local alignment search tool), where a query sequence was compared to a set of reference sequences of known genotypes. The query sequence was broken into segments for comparison to the reference so that the mosaic organization of recombinant sequences could be revealed (Reuman et al. 2010).

### Prediction of HBV subtypes

DNA sequences were translated to proteins using internet software expaXy translation tools. The best and high scoring matches with HBV proteins were aligned with CLUSTAL W software program (Larkin et al. 2007), and the alignment was edited by BioEdit sequence alignment program (Hall 1999). Amino acid alterations within the small S portion of surface protein were determined by comparing with reference sequences available in data bank (Additional file 2). HBV subtypes were determined according to the presence of amino acid residues at specific locations on surface proteins. The subtypes were determined by analyzing amino acid positions, like at position 122 (Lys/Arg for subtype d/y determinant), 160 (Lys/Arg for w/r), and 127 (Pro/Thr/Leu-Ile for w1-2/w3/w4) (Magnius and Norder 1995). Amino acid positions 134 and 159 were used to discriminate between type *ayw*1 and *ayw*2.

### Accession numbers

After analyzing the HBV sequence**s,** we submitted our sequences to GenBank and we got an accession number for each sequence. We had total 39 sequences and the accession numbers are sequentially organized from KF498977 to KF499015.

## Results

**Fig. 1:**
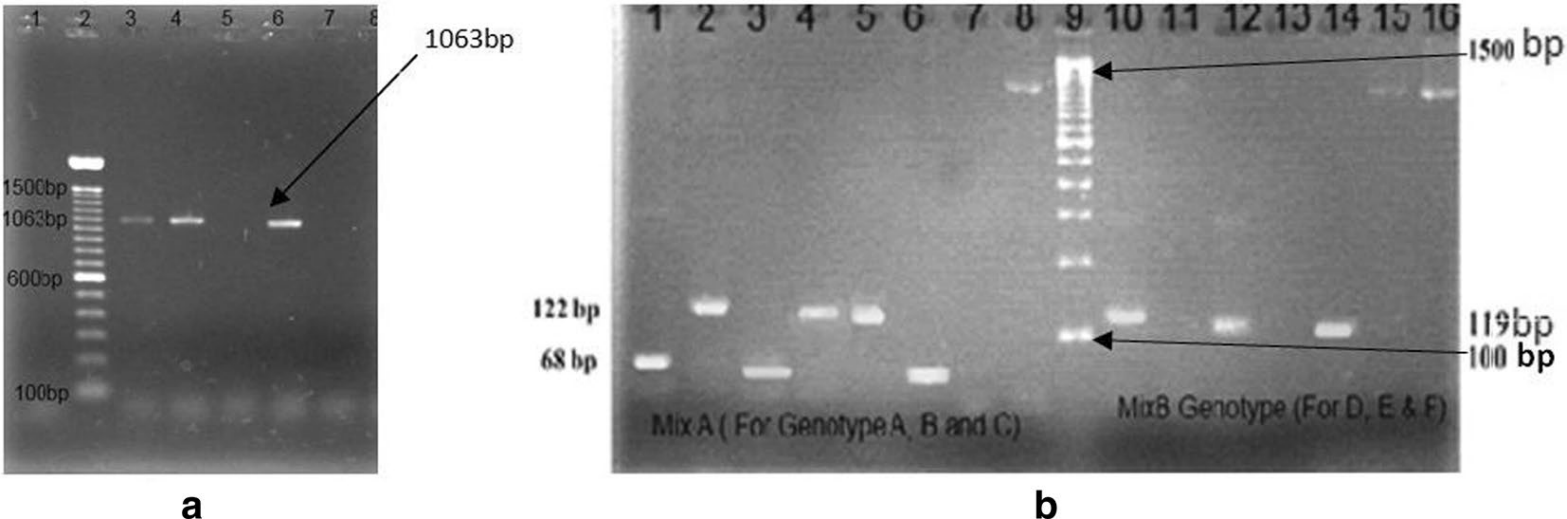
Agarose gel electrophoresis. **a** Showing PCR positive band size of 1063 bp of HBV Large S gene. **b** The typical electrophoresis patterns using type specific primer of different HBV genotypes (Genotype, A-68 bp; C-122 bp; D-119 bp). In this figure, *lane 1*, *2*, *3*, *4*, *5* and *6* showed the band for the sample accession number of KF499015, KF498982, KF498984, KF498996, KF499007 and KF498990 respectively. *Lane 7* was mix A negative control whereas *lane 15* was negative control for mix B. The same samples were loaded sequentially from *lane 10*–*14* and *lane 16* contains KF498990. (The figure was cropped to minimize the figure size and included the numerical values as well as labels)

The 50 patients, included in this study, diagnosed as chronic carrier by physicians. The DNA levels were previously measured for all patients using real time PCR (HBV TaqMan, Roche), had a value ranged from 3.1 × 10^4^ to 1.2 × 10^12^ copies/ml. However, in EIA test we found 47 of them were HBsAg positive and 3 were negative. When amplified with P1/S1-2 primers, a1063 bp of amplified PCR product was revealed on agarose gel electrophoresis (Fig. 1a). After that, 39 of 50 PCR positive samples were selected from for direct sequencing. Among them 38 were HBsAg positive and one was HBsAg negative (Additional file 3). The samples showing weak bands or multiple bands were excluded from sequencing as well as from others study. Two of the three HBsAg negative samples were also excluded due to showing weak bands. Next, the samples were subjected to nested PCR using genotype specific primers.

### Genotyping and sub genotyping by using DNA sequence

**Fig. 2:**
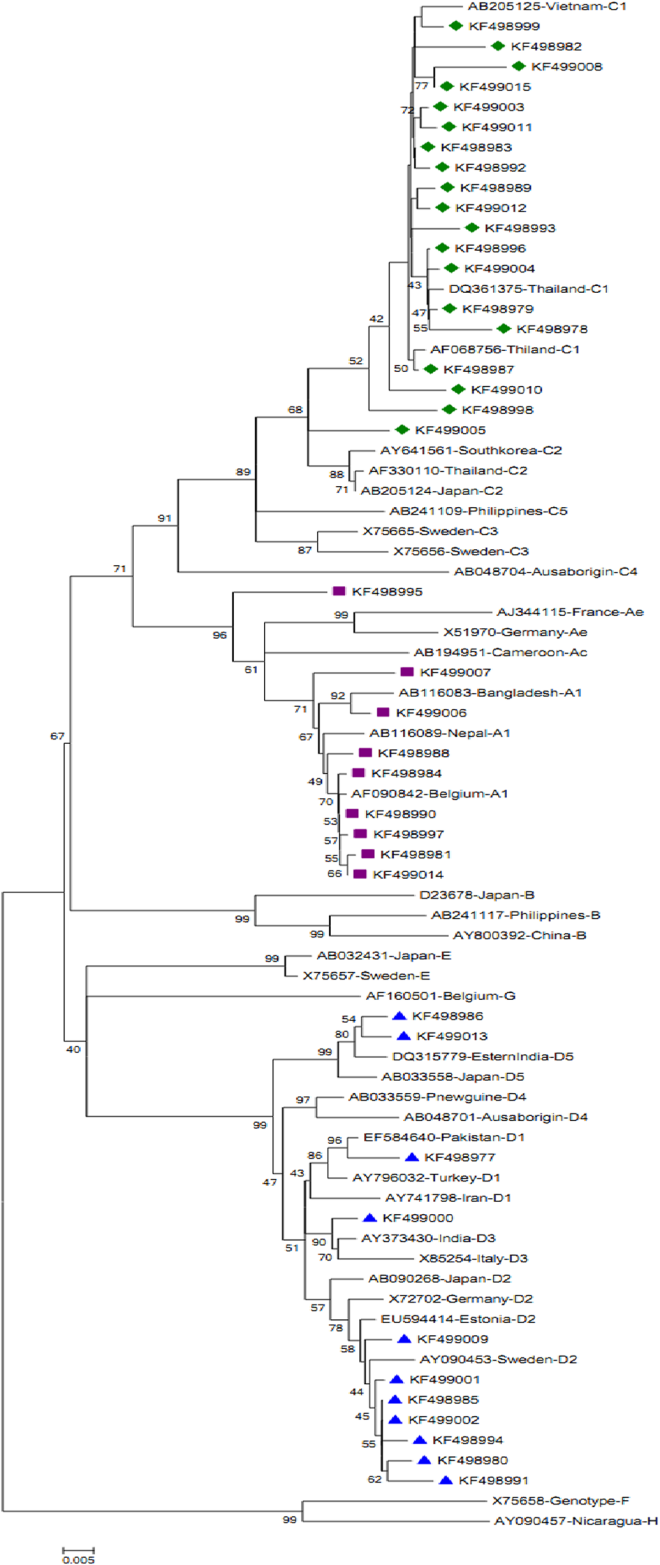
Phylogenetic Tree. The tree is constructed using 950 bp nucleotide sequence (2908-643 bp of complete referance genome) of Pre-S1/Pre-S2/S region of HBV. Referance genome of genetypes A to H representing the standard HBV genotypes throughout the world were used for analysis (Additional file 2).* Bootstrap values* (≥40) are indicated at the nodes of the brance

Genotyping of 39 HBV isolates (Accession number; [GenBank: KF498977-KF499015]) were determined by constructing phylogenetic tree (Fig. 2). The test sequences were grouped with the reference sequences (Additional file 2) according to their genotypes. The predominant genotypes were genotypes C (48.7 %), D (28.2 %) and A (23.1 %). The genotypes of these sequences were also determined by NCBI genotyping tools which gave complete fidelity with the phylogenetic result.

During the determination of subgenotypes, we found only single subgenotype within the genomic group C and genotype A. These were C1 and A1 respectively. However, in case of genotype D, multiple subgenotypes were found (Table 1). These were D1, D2, D3 and D5.

**Table 1:** Distribution of genotypes and subgenotypes of HBV isolates

Genotype	SubGenotype	Total	Percentage
A	A1	9	23.1
C	C1	19	48.7
D	D1	1	2.5
	D2	7	18
	D3	1	2.5
	D5	2	5.1
A and C		2	5.1
A and D		3	7.6

Genotypes and subgenotypes were determined by phylogenetic tree, PCR system and NCBI genotyping tool

### Genotyping by genotype-specific primers

In this method, the genotypes were determined by observing the genotype specific band on the agarose gel electrophoresis. The samples giving 1063 bp PCR product in first round PCR by P1/S-2 primers were subjected to multiplex PCR using type-specific primers (Fig. 1b). Here, 17 of 39 samples were successfully analyzed, rest of the samples did not produce right sized product or no product at all. Three of 17 samples were detected as genotype A, 6 were genotype C and 4 were genotype D. However, for some cases we found more than one band for a single sample, implied that those patients carried multiple genotypes or mix infection. In Fig. 1b, lane 1 and lane 10 showed band from single sample (KF499015) which indicates, this sample carried both genotype A (68 bp on mix A) and D (119 bp in mix B). Likewise, KF499007 contained both genotype C and D (122 bp band in mix A in lane 5 and 119 bp band in mix B in lane 14). In our experiment, we found mixed genotypes A and D in 3 samples as well as genotype C and D in 2 samples.

### Distribution of subtype

Amino acid substitutions in residues related to subtype specificity were determined for all 39 samples. The predominant subtypes we found in our experiment were, *adr* (41 %), *adw*2 (28.2 %), *ayw*2 (5.1 %) and *ayw*3 (25.6 %) (Table 2).

**Table 2:** Distribution of subtypes of HBV isolates (Additional file 4)

Subtype	Amino acids at specific position	Total	Percentage	Sub genotypes
*adw*2	122K + 127P + 160K	11	28.2	A1 (8)
				C1 (3)
*ayw*3	122R + 127T + 160K	10	25.6	A1 (1)
				D5 (2)
				D2 (7)
*adr*	122K + 127P + 160R	16	41	C1 (16)
*ayw*2	122R + 127P + 140T + 159G + 160K	2	5.1	D1 (1)
				D3 (1)
Total		39	100	

The subtypes was determined by presence of specific amino acids at a paticular position as described in ref 5 and 7

### Amino acid substitutions in surface protein

Mutation on surface gene lead to altered conformation of the protein in several ways. In our study we have detected several amino acid substitutions on S region in some isolates. Four of the 39 isolates showed amino acid substitutions in a total of 5 positions. Sample 2-P2 [GenBank: KF498978] showed two amino acid substitutions at G119R and P120A, HBV-350 [GenBank: KF498986] at G145R, HBV-418 [GenBank: KF498987] at C149 W and HBV-532 [GenBank: KF498998] at S117I; located within the major hydrophilic region (MHR) of the S gene and few are within the ‘a’ determinant. The correlation between the amino acid alteration and course of disease pattern had not been examined in this study.

## Discussion

In recent years viral hepatitis is a major public health problem worldwide. As with other Asian countries, Bangladesh is also hyper-endemic for HBV related liver disease, with seroprevalence of HBsAg is 6–7 % (Rahman et al. 2007). However, data on genotype or subtype of HBV are not sufficient. Some previous studies were conducted based on the serological assay where they have only shown the sero-prevalence HBV in Bangladesh (Rahman et al. 2007; Zaki et al. 2003). In this study, we have tried to find out the distribution of genotypes and subtypes in Bangladesh, because, genotyping and subtyping of HBV has its great implementation as HBV have their characteristic geographical distribution. Determination of genotypes and subtypes also has clinical as well as therapeutic importance as vaccine development, drug selection, and mutant identification (Shi 2012). In Bangladesh, we have provided the first molecular study on HBV. We sequenced and analyzed the large surface gene of HBV (spanning from pre-S1, pre-S2 and most part of small surface gene) and the genotypes were determined by phylogenetic tree (by comparing with reference strains of Additional file 2) using Mega5 software (Tamura et al. 2007). In this study we also determine HBV genotypes by NCBI genotyping tools. This study showed that the genotypes C, D and A are circulating in the country. Similar results have been published from the ‘Eastern India’, close neighbor of Bangladesh, where in addition to genotype A and D, genotype C is prevalent (Datta et al. 2008a, b; Datta 2008). When the genotyping was done by PCR using type-specific primers, similar pattern of genotypes were found. Although some of the samples (11.76 %) contained mixture of genotypes; A/D and C/D, indicating that PCR based genotype is more useful. This is also convenient and cost effective, but till now it only able to detect the genotypes A–F. The other genotypes (G, H, I and J) might be missed in this method, and so, it might not reveal the correct picture of HBV genotype distribution. On the other hand, the first two methods are unable to determine the multiple genotypes present in the same patient. However, we got approximately same result in three genotyping systems (without considering the mixed genotypes which found PCR based genotyping system).

Analysis of phylogenetic tree also revealed that genotype C was found to be clustered only with the reference sequences of Vietnam and Thailand (Phung et al. 2010). Genotype D and A showed different clustering pattern. In this study, genotype A was clustered with reference sequences from Japan, Pakistan, India, Turkey, Italy and Iran (Mohebbi et al. 2008), whereas, genotype D showed the different pattern of branch and was not only with Asian reference sequences but also with European references sequences. Prevalence of subgenotypes of above mentioned three genotypes were also determined. Genotype A (23.1 %) and C (48.7 %) were confined only within the sub-genotype A1 and C1 respectively. On the other hand, genotype D (28.2 %) had different sub-genotype. These were D1 (2.5 %), D2 (18 %), D3 (2.5) and D5 (5.1 %).

To determine the subtype, all of the nucleotide sequences were translated into amino acid sequences. We found that the prevalent subtypes were *adr* (41 %), followed by *adw*2 (28.2 %), *ayw*3 (25.6 %) and *ayw*2 (5.1 %). The subtype *adr* completely restricted on genomic group C. However, the subtype *adw*2 found within the genotype A and C. The subtype *ayw* were mostly found among the genotype D.

Substitutions of amino acids in the surface protein were also well documented in our study. All of them were within the MHR of the S gene. Mutation at G145R (responsible for immune escape mutant), reported earlier (Waters et al. 1992) was observed in one of the cases (HBV-350 [GenBank: KF498986]). This mutation might be the cause of HBsAg negativity of that sample, although it gave PCR positive result. A number of other mutations on HBV S gene have also been reported (Bian et al. 2013; Avellon and Eschevarria 2006). A variation of S117I/N/T/C/G was reported by Avellon and Eschevarria (2006). In our study, Serine in this position has been found to be replaced by Isoleucine in one case (HBV-532 [GenBank: KF498998]). Detail studies on the impact of HBV surface gene mutations on its structure and the course of pathogenesis could not performed.

Immunization program against HBV in Bangladesh have been started since 2004 (Rashid and Rafiq 2006). By this time the viruses might able to manage their structural changes to escape vaccination. However, people of Bangladesh are still not very conscious about HBV related liver infections. Though national immunization program has been launched only recently, however, post vaccination surveillance has not been started to confirm whether the vaccine worked properly or whether vaccinated children truly developed anti-HBV antibody. Therefore, a detailed study is essential with large number of sample volume to gather result about genotype, subtypes, subgenotypes and immune escape mutation rate of HBV in Bangladesh.

## Conclusion

To know about the commonly found genotypic and subtypes of HBV in Bangladesh this article will give a clear idea, which is very important for both national and international virologist.

## Additional files

10.1186/s40064-016-1840-2 Type specific primers used to determine genotype A-F.
10.1186/s40064-016-1840-2 Accession numbers used as reference sequence.
10.1186/s40064-016-1840-2 Details information of the 39 HBV isolates which were randomly selected from 50 PCR positive samples.
10.1186/s40064-016-1840-2 Alignment of all proteins.

## Abbreviations

HBV: hepatitis B virus
PCR: polymerase chain reaction
LC: liver cirrhosis
HCC: hepatocellular carcinoma
DNA: deoxy ribonucleic acid
RNA: ribonucleic acid
EIA: enzyme immune assay
HBsAg: hepatitis B surface antigen
HBeAg: HBe antigen
anti-HBe: HBe antibody
NCBI: National Center for Biotechnology Information
BLAST: basic local alignment search tool
bp: base pair
MHR: major hydrophilic region
