# Supplementary material for: Prevalence of genotypes and subtypes of hepatitis B viruses in Bangladeshi population
Source: Springerplus. 2016 Mar 5;5:278. doi: 10.1186/s40064-016-1840-2 (PMC4779089; doi:10.1186/s40064-016-1840-2)
Supplement: Supplementary file 1 — 10.1186/s40064-016-1840-2 Type specific primers used to determine genotype A-F. [file 40064_2016_1840_MOESM1_ESM.docx]

**Additional file 1: Type specific primers used to determine genotype A-F**

| **Type** | **Primer Name** | **Sequences (position, speciﬁcity)** |
| --- | --- | --- |
| Sense Primer for genotype A to E | B2 | 5ʹ-GGC TCM AGT TCM GGA ACA GT-3ʹ |
|  |  | (nt 67–86, types A to E speciﬁc) |
| Antisense Primer (Mix A) | BA1R | 5ʹ-CTC GCG GAG ATT GAC GAG ATG T-3ʹ |
|  |  | (nt 113–134, type A speciﬁc) |
|  | BB1R | 5ʹ-CAG GTT GGT GAG TGA CTG GAG A-3ʹ |
|  |  | (nt 324–345, type B speciﬁc) |
|  | BC1R | 5ʹ-GGT CCT AGG AAT CCT GAT GTT G-3ʹ |
|  |  | (nt 165–186, type C speciﬁc) |
| Antisense Primer for genotype D to F | B2R | 5ʹ-GGA GGC GGA TYT GCT GGC AA-3ʹ |
|  |  | (nt 3078–3097, types D to F speciﬁc) |
| Sense Primers (Mix B) | BD1 | 5ʹ-GCC AAC AAG GTA GGA GCT-3ʹ |
|  |  | (nt 2979–2996, type D speciﬁc |
|  | BE1 | 5ʹ-CAC CAG AAA TCC AGA TTG GGA CCA-3ʹ |
|  |  | (nt 2955–2978, type E speciﬁc) |
|  | BF1 | 5ʹ-GYT ACG GTC CAG GGT TAC CA-3ʹ |
|  |  | (nt 3032–3051, type F speciﬁc) |

The above listed primers were used to determine the HBV genotypes (A-F). That is why these primers are mentioned as ‘type specific primers’. Details has been described on materials and method section
