# Supplementary material for: Prevalence of genotypes and subtypes of hepatitis B viruses in Bangladeshi population
Source: Springerplus. 2016 Mar 5;5:278. doi: 10.1186/s40064-016-1840-2 (PMC4779089; doi:10.1186/s40064-016-1840-2)
Supplement: Supplementary file 2 — 10.1186/s40064-016-1840-2 Accession numbers used as reference sequence. [file 40064_2016_1840_MOESM2_ESM.docx]

**Additional file 2: Accession numbers used as reference sequence**

| DNA Analysis | | Protein Analysis | |
| --- | --- | --- | --- |
| **Accession Number** | **Accession Number** | **Accession Number** | **Accession Number** |
| GenBank: AB116083 | GenBank: AY741798 | GenBank: AB116083 | GenBank: AHC69877 |
| GenBank: AF090842 | GenBank: AB090268 | GenBank: ADV77063 | GenBank: AHC69905 |
| GenBank: AB116089 | GenBank: AY090453 | GenBank: ADB03191 | GenBank: AHC69843 |
| GenBank: AB194951 | GenBank: EU594414 | GenBank: ADV77063 | GenBank: AHC69850 |
| GenBank: AJ344115 | GenBank: X72702 | GenBank: AF068756 | GenBank: AHC69882 |
| GenBank: X51970 | GenBank: AY373430 | GenBank: DQ361375 | GenBank: AHC69891 |
| GenBank: AB241117 | GenBank: X85254 | GenBank: AB205125 |  |
| GenBank: AY800392 | GenBank: AB033559 | GenBank: AY741798 |  |
| GenBank: D23678 | GenBank: AB048701 | GenBank: EF584640 |  |
| GenBank: AB241109 | GenBank: DQ315779 | GenBank: AY796032 |  |
| GenBank: AB048704 | GenBank: AB033558 | GenBank: AY653888 |  |
| GenBank: X75665 | GenBank: AB032431 | GenBank: ACF95240 |  |
| GenBank: X75656 | GenBank: X75657 | GenBank: ACF95287 |  |
| GenBank: DQ361375 | GenBank: X75658 | GenBank: ABD36979 |  |
| GenBank: AB205125 | GenBank: AF160501 | GenBank: ACG68716 |  |
| GenBank: AF068756 | GenBank: AY090457 | GenBank: ACX70039 |  |
| GenBank: AY641561 | GenBank: X02763 | GenBank: AAQ19248 |  |
| GenBank: AF330110 | GenBank: D00329 | GenBank: AAQ19256 |  |
| GenBank: AB205124 | GenBank: X04615 | GenBank: AFH02531 |  |
| GenBank: EF584640 | GenBank: X65259 | GenBank: AHC69836 |  |
| GenBank: AY796032 | GenBank: X75657 | GenBank: AHC69863 |  |
| GenBank: AF160501 | GenBank: X69798 | GenBank: AHC69870 |  |
| GenBank: AY090454 |  | GenBank: AHC69898 |  |

The above listed GenBank accession numbers were used as a reference sequences. To analyze our DNA sequences, we used GenBank accession numbers of first two columns (showing DNA analysis). The accession numbers of last two columns (showing protein analysis) were used to analysis our protein sequences.
