# Supplementary material for: Prevalence of genotypes and subtypes of hepatitis B viruses in Bangladeshi population
Source: Springerplus. 2016 Mar 5;5:278. doi: 10.1186/s40064-016-1840-2 (PMC4779089; doi:10.1186/s40064-016-1840-2)
Supplement: Supplementary file 3 — 10.1186/s40064-016-1840-2 Details information of the 39 HBV isolates which were randomly selected from 50 PCR positive samples. [file 40064_2016_1840_MOESM3_ESM.docx]

**Additional file 3: Details information of the 39 HBV isolates which were randomly selected from 50 PCR positive samples**

| **Accession No.** | **Sample ID** | **DNA Copies/ml** | **Age** | **Sex** | **HBeAg** | **HBsAg** | **PCR Genotyping** | **Sub genotype** | **Subtype** |
| --- | --- | --- | --- | --- | --- | --- | --- | --- | --- |
| KF498977 | HBV-E-15 | NI | 27 | F | ND | +ve | ND | D1 | ayw2 |
| KF498978 | HBV-2-P2 | 2.3x 10^8^ | 39 | M | +ve | +ve | ND | C1 | adr |
| KF498979 | HBV-1-4 | 1.5x10^9^ | 43 | M | +ve | +ve | ND | C1 | adr |
| KF498980 | HBV-6-6 | 1.4x10^7^ | 37 | M | -ve | +ve | ND | D2 | ayw3 |
| KF498981 | HBV-7-7 | 2.4x10^7^ | 46 | M | +ve | +ve | ND | A1 | adw2 |
| KF498982 | HBV-142 | 2.6x10^11^ | 30 | M | -ve | +ve | Done | C1 | adr |
| KF498983 | HBV-157 | 1.7x10^12^ | 22 | M | +ve | +ve | ND | C1 | adr |
| KF498984 | HBV-180 | 3x10^12^ | 28 | M | +ve | +ve | Done | A1 | adw2 |
| KF498985 | HBV-272 | 2.4x10^12^ | 25 | F | ND | +ve | Done | D2 | ayw3 |
| KF498986 | HBV-350 | 6.3x10^6^ | 20 | F | -ve | -ve | Done | D5 | ayw3 |
| KF498987 | HBV-418 | 2.3x10^7^ | 52 | F | -ve | +ve | ND | C1 | adr |
| KF498988 | HBV-421 | 2.9x10^7^ | 20 | M | +ve | +ve | ND | A1 | adw2 |
| KF498989 | HBV-422 | 6.8x10^9^ | 26 | M | -ve | +ve | ND | C1 | adr |
| KF498990 | HBV-430 | 2.9x10^7^ | 38 | M | +ve | +ve | ND | A1 | adw2 |
| KF498991 | HBV-435 | 1.2x10^7^ | 25 | M | ND | +ve | ND | D2 | ayw3 |
| KF498992 | HBV-439 | 5.8x10^12^ | 51 | M | -ve | +ve | ND | C1 | adr |
| KF498993 | HBV-453 | 1.6x10^7^ | 28 | M | ND | +ve | ND | C1 | adw2 |
| KF498994 | HBV-499 | 1.4x10^9^ | 34 | F | -ve | +ve | ND | D2 | ayw3 |
| KF498995 | HBV-506 | 5.7x10^11^ | 25 | F | -ve | +ve | ND | A1 | ayw3 |
| KF498996 | HBV-507 | 1.2x10^12^ | 24 | M | +ve | +ve | Done | C1 | adr |
| KF498997 | HBV-526 | 2x10^12^ | 26 | M | +ve | +ve | ND | A1 | adw2 |
| KF498998 | HBV-532 | 5.1x10^12^ | 30 | M | +ve | +ve | Done | C1 | adr |
| KF498999 | HBV-553 | 4.2x10^12^ | 30 | M | +ve | +ve | ND | C1 | adr |
| KF499000 | HBV-554 | 2.2x10^12^ | 9 | M | +ve | +ve | ND | D1 | ayw2 |
| KF499001 | HBV-559 | 2.3x10^12^ | 65 | M | +ve | +ve | ND | D2 | ayw3 |
| KF499002 | HBV-568 | 5.6x10^9^ | 30 | M | +ve | +ve | ND | D2 | ayw3 |
| KF499003 | HBV-5-685 | 7.8x10^7^ | 28 | F | ND | +ve | ND | C1 | adr |
| KF499004 | HBV-845 | NI | 34 | M | ND | +ve | ND | C1 | adw2 |
| KF499005 | HBV-13890 | 1.5x10^9^ | 20 | M | +ve | +ve | Done | C1 | adr |
| KF499006 | HBV-13915 | 7.5x10^6^ | 26 | M | -ve | +ve | Done | A1 | adw2 |
| KF499007 | HBV-13925 | 4.2x107 | 35 | M | +ve | +ve | Done | A1 | adw2 |
| KF499008 | HBV-13944 | NI | 60 | M | -ve | +ve | Done | C1 | adr |
| KF499009 | HBV-13952 | 2.1x10^11^ | 8 | F | +ve | +ve | Done | D2 | ayw3 |
| KF499010 | HBV-13961 | 7.5x10^6^ | 65 | M | -ve | +ve | Done | C1 | adw2 |
| KF499011 | HBV-13962 | 9.9x10^6^ | 26 | M | +ve | +ve | Done | C1 | adr |
| KF499012 | HBV-13982 | 6.2x10^6^ | 17 | M | ND | +ve | Done | C1 | adr |
| KF499013 | HBV-14008 | 5.2x10^12^ | 20 | M | +ve | +ve | Done | D5 | ayw3 |
| KF499014 | HBV-14027 | 3.1x10^6^ | 29 | M | ND | +ve | Done | A1 | adw2 |
| KF499015 | HBV-14028 | 2.8x10^7^ | 11 | M | +ve | +ve | Done | C1 | adr |

Abbreviations: M=Male; F=Female; +ve=Positive; -ve=Negative; NI= No information; ND=Not done
