# Supplementary material for: Prevalence of genotypes and subtypes of hepatitis B viruses in Bangladeshi population
Source: Springerplus. 2016 Mar 5;5:278. doi: 10.1186/s40064-016-1840-2 (PMC4779089; doi:10.1186/s40064-016-1840-2)
Supplement: Supplementary file 4 — 10.1186/s40064-016-1840-2 Alignment of all proteins. [file 40064_2016_1840_MOESM4_ESM.rtf]

                       
Additional File 4a. Alignment of all proteins (small S portion) 
          
                            10        20        30        40        50        60        70        80        90       100                  
                   ....|....|....|....|....|....|....|....|....|....|....|....|....|....|....|....|....|....|....|....|
Standard           MESTTSGFLGPLLVLQAGFFLLTRILTIPQSLDSWWTSLNFLGGAPTCPGQNLQSPTSNHSPTSCPPICPGYRWMCLRRFIIFLFILQLCLIFLLVLLDY 
KF498977           ..NI...L....................................TTV.L...S..............T...................L............ 
KF498978           ...................................................KF..................................L............ 
KF498979           .......................................................................................L............ 
KF498980           ..NI...................................K....TTV.L...S..............T...................L............ 
KF498981           ..NI........................................S.V.L...S..................................L............ 
KF498982           .......................................................................................L............ 
KF498983           .......................................................................................L............ 
KF498984           ..NI........................................S.V.L...S..................................L............ 
KF498985           ..NI........................................TTV.L...S..............T...................L............ 
KF498986           ..NI........................................TTV.L...S..............A...................L............ 
KF498987           .......................................................................................L............ 
KF498988           ..NI........................................S.V.L...S..................................L............ 
KF498989           .......................................................................................L............ 
KF498990           ..NI........................................S.V.L...S..................................L............ 
KF498991           ..NI........................................NTV.L...S..............T...................L............ 
KF498992           .......................................................................................L............ 
KF498993           .............................R................K........................................L............ 
KF498994           ..NI........................................TTV.L...S..............T...................L............ 
KF498995           ..NI........................................S.V.L...S..............T...................L............ 
KF498996           .......................................................................................L............ 
KF498997           ..NI........................................S.V.L...S..................................L............ 
KF498998           .......................................................................................L............ 
KF498999           ....A..................................................................................L............ 
KF499000           ..NI........................................TTV.L...S..............T...................L............ 
KF499001           ..NI........................................TTV.L...S..............T...................L............ 
KF499002           ..NI........................................TTV.L...S..............T...................L............ 
KF499003           .......................................................................................L............ 
KF499004           .......................................................................................L............ 
KF499005           ..NI........................................S.V.L...S..................................L............ 
KF499006           ..NI........................................S.V.L...S..................................L............ 
KF499007           ..NI..........................................V.R...S..................................L............ 
KF499008           .......................................................................................L.....S...... 
KF499009           ..NI........................................TTV.L...S..............T...................L............ 
KF499010           ..............................................A.....S...............S..................L............ 
KF499011           .......................................................................................L............ 
KF499012           .......................................................................................L............ 
KF499013           ..NI........................................TTV.L...S..............T...................L............ 
KF499014           ..NI........................................S.V.L...S..................................L............ 
KF499015           .......................................................................................L.....S...... 
Clustal Consensus  **. :**:*********************:*********:**** . * **: ************** .****************** ***** ****** 


                           110       120       130       140       150       160       170       180       
                   ....|....|....|....|....|....|....|....|....|....|....|....|....|....|....|....|....
Standard           QGMLPVCPLLPGTSTTSTGPCKTCTIPAQGTSMFPSCCCTKPSDGNCTCIPIPSSWAFARFLW--------------------- 
KF498977           .........I..S........R...T.......Y..................A.....GK...EW------------------- 
KF498978           ..................RA...........................................EW------------------- 
KF498979           ...............................................................EW------------------- 
KF498980           .........I..S....V...R...TTV.....Y........................GK...E-------------------- 
KF498981           .........I..ST...........T....N...........T................K...EW------------------- 
KF498982           ...............................................................EWASVRFSWLSLLVPFVQWF- 
KF498983           ...............................................................E-------------------- 
KF498984           .........I..ST...........T....N...........T.................Y----------------------- 
KF498985           .........I..S....V...R...TTV.....Y....................A...GK...EW------------------- 
KF498986           .........I..S........R..MTT......Y..........R.....L.......GK..---------------------- 
KF498987           ................................................W..............EWASV-VSPGSFLVPLFML-- 
KF498988           .........I..ST...........T....N...........T................KY..EWASVRFSWLSLLVPFVQWF- 
KF498989           ...............................................................EWASVRFSWLSLLVPFVQWF- 
KF498990           .........I..ST...........T....N...........T................KY..EWASVRFSWLSLLVPFVQWF- 
KF498991           .........I..S....V...R...TTV.....Y........................GK...EWASARFSWLSLLVPFVQWF- 
KF498992           .......................................................S......RE-------------------- 
KF498993           ...........................................................KY----------------------- 
KF498994           .........I..S....V...R...TTV.....Y........................GK...EWASARFSWLSLLVPFVQWFD 
KF498995           .........I..S....V...R...TTV.....Y........................GK...EWASARFSWLSLLVPFVQWFD 
KF498996           .......................................................S.....----------------------- 
KF498997           .........I..ST...........T....N...........T............S...KY..--------------------- 
KF498998           ................I..............................................EWASIRFSWLSLLVPFVQWF- 
KF498999           ...............................................................EWASVRFSWLSLLVPFVQWF- 
KF499000           .........I..S........R...T.......Y........................GK...EWASARFSWLSLLVPFVQWF- 
KF499001           .........I..S....V...R...TTV.....Y........................GK...EWASARFSWLSLLVPFVQWF- 
KF499002           .........I..S....V...R...TTV.....Y.....................S..GKY.P--------------------- 
KF499003           ...............................................................EW------------------- 
KF499004           ...........................................................K...EW------------------- 
KF499005           ...............................................................EWASVRFSWLSLLVPFVQWF- 
KF499006           .........I..ST...........T....N...........T...............GKY..EW------------------- 
KF499007           .........I..ST...........T....N...........T................KY..--------------------- 
KF499008           R..............................................................EWASVRFSWLSLLVPFVQWF- 
KF499009           .........I..S....V...R...TTV.....Y........................GK...EW------------------- 
KF499010           ...............................Y......S...T................KY..--------------------- 
KF499011           ............................................................Y.---------------------- 
KF499012           ..-......................T..................................Y----------------------- 
KF499013           .........I..S........R..MTT......Y........................GK..---------------------- 
KF499014           .........I..ST...........T....N...........T................KY..EWASVRFSWLSLLVPFVQWF- 
KF499015           R..............................................................EWASVRFSWLSLLVPFVQWF- 
Clustal Consensus  :* ******:**::** . .*:**  ..**. *:****.***:* *** * *.*: **.::                        


Additional File 4b. Alignment of Large S protein      
                             10        20        30        40        50        60        70        80        90       100                  
                            ....|....|....|....|....|....|....|....|....|....|....|....|....|....|....|....|....|....|....|....|
gi|59418|emb|X02763.1       MGGWSSKPRKGMGTNLSVPNPLGFFPDHQLDPAFGANSNNPDWDFNPVKDDWPAANQVGVGAFGPRLTPPHGGILGWSPQAQGILTTVSTIPPPASTNRQ 
gi|221497|dbj|D00329.1|     ..................................K...E.....L..H..N..D.HK........GF......L............S.PAA......... 
gi|59585|emb|X04615.1|      .........Q.....................................N..H..E.....A.....GF......L.............LPAA......... 
gi|59439|emb|X65259.1|      -----------..Q...TS...............R..TA........N..T..D..K..A....LGF......L...........H..PAN......... 
gi|452617|emb|X75657.1|     -M.L.WTVPLEW.K.I.TT...............R..TR.....H..N..H.TE..K........GF......L.........M.K.LPAD......... 
gi|59422|emb|X69798.1|      ..APL.TT.R...Q..................L.R...SS......TN..S..M..K....GY..GF......L.........V...LPAD........R 
gi|6983934|gb|AF160501.1|   -M.L.WTVPLEW.K...AS.....L.........R..T.........K..P..E..K.....Y..GF......L......S..T...LPAD......... 
gi|22135696|gb|AY090454.1|  ..APL.TA.R...Q..................L.R...SS......TN..N..M..K....G...GF......L.............SPPD........R 
KF498977                    --------------------..............R..TA........N..T..D..K..A....LGF......L...........Q.LPAN......... 
KF498978                    --------------------......G....................N..Q..........S...GF.....NL..............PAA......... 
KF498979                    --------------------......G....................N..Q..........S...GF.....NL..............PAA.....S... 
KF498980                    --------------------..............R..TA........N..T..D..K..A....LGF......L..........IQ.LPAN......... 
KF498981                    --------------------...........................I..H..Q...........GF......V...........A..P.V......... 
KF498982                    ------------------------------------------C..K.N..Q.SVP......S...GF.....SL..............PAA......... 
KF498983                    ---------------......V....G....................N..Q..........S...GF.....SL..............PAA......... 
KF498984                    ------------------------...........S...........I..H..Q...........GF......V...........A..PAV......... 
KF498985                    -------------------------------------TA........N..T..D..K..A....LGF......L..........IQ.LPAN......... 
KF498986                    ------------------------------------------.....N..T..D..K..A....LGF......L...........Q.LPAN....A.... 
KF498987                    ----------------------------------------.......N..Q..........S...GF.....SL..............PAA......... 
KF498988                    ------------------------------.................I..H..Q...........GF..................A..PAV......... 
KF498989                    ----------------------------...................K..Q..........S...GF.....SL..............PAA......... 
KF498990                    ----------------------------------------.......I..H..Q...........GF......V...........A..PAV......... 
KF498991                    -----------------------------------------......N..T..D..K..A....LGF......L..........IQ.LPAN......... 
KF498992                    ------------------------------.................N..Q..........S...GF.....SL..............PAA......... 
KF498993                    ----------------------A...G..V.................N..Q..............GF.....NL.............MPAA......... 
KF498994                    --------------------------------..R..TA........N..T..D..K..A....LGF......L..........IQ.LPVN......... 
KF498995                    -----------------------------..................I..H..Q...........GF......V.........L.A..PAV......... 
KF498996                    ---------------------------....................N..Q..........S...GF.....NL..............PAA......... 
KF498997                    --------------------...........................I..H..Q...........GF......V...........A..PAV...V..... 
KF498998                    ....Y...SQ......C..........L.....L.............N..Q........A.....GF......L.............LPAA......... 
KF498999                    --------------------------G....................N..Q..........S...GF.....SL..............PAA......... 
KF499000                    ------------------------..........R..TA........N..T..D..K..A....LGF......L...........Q.LPAN......... 
KF499001                    ------------------------..........R..TA........N..T..D..K..A....LGF......L..........IQ.LPAN......... 
KF499002                    -----------------------------------------......N..T..D..K..A....LGF......L..........IQ.LPAN......... 
KF499003                    --------------------......G....................N..Q..........S...GF.....SL..............PAA......... 
KF499004                    --------------------......G....................N..Q..........S...GF.....NL..............PAA......... 
KF499005                    -----------------------------...............L..N..Q..........S...GF......L..............PAA......... 
KF499006                    -------------------............................I..H..Q...........GF......V...........A..PAV......... 
KF499007                    ------------..................................TI..H..Q...........GF......V...........A..PAV......... 
KF499008                    --------------------------G....................N..Q..........S...GF.....SL.........T....PAA......... 
KF499009                    ---------------------------------------........N..T..E..K..A....LGF......L..........IQ.LPAN......... 
KF499010                    -----------------------------------....D.......N..Q..........S...GF.....SL..............PAA......... 
KF499011                    --------------------------G....................N..Q..........S...GF.....SL..............PAA......... 
KF499012                    --------------------------------...............N..Q..........S...GF.....NL..............PAA......... 
KF499013                    ---------------------------------------........N..T..D..K..A....LGF......L...........Q.LPAN....A.... 
KF499014                    --------------------------.....................I..H..Q...........GF......V...........A..P.V......... 
KF499015                    -----------------------------..................N..Q..........S...GF.....SL..............PAA......... 
Clustal Consensus                                                      * :. ** *. .::**.*.:*  :*****.:******:** : : .  ***.::**: 

                                    110       120       130       140       150       160       170       180       190       200         
                            ....|....|....|....|....|....|....|....|....|....|....|....|....|....|....|....|....|....|....|....|
gi|59418|emb|X02763.1       SGRQPTPISPPLRDSHPQAMQWNSTAFHQTLQDPRVRGLYLPAGGSSSGTVNPAPNIASHISSISARTGDPVTNMENITSGFLGPLLVLQAGFFLLTRIL 
gi|221497|dbj|D00329.1|     .......L......T..........T...........A..F..........S..Q.TV.A....LSK.....P.....A..L...............K.. 
gi|59585|emb|X04615.1|      .........................T...A.L........F............V.TT..P....FS.....AP...ST...................... 
gi|59439|emb|X65259.1|      .......L.....NT..........T..............F............VLTT..PL..........AL........................... 
gi|452617|emb|X75657.1|     ........T.....T..........T...A..........F............V.TT..L....FS.I...AP...S....................K.. 
gi|59422|emb|X69798.1|      ...K...V......T..........Q...A.L.....A..F.........Q....T...LT...FSK..G.AM..D.....L.........VC....K.. 
gi|6983934|gb|AF160501.1|   .............................A..N.K.....F........I...V.T........FS.I...AP........................... 
gi|22135696|gb|AY090454.1|  ...K...V......T..........Q...A.L........F.......E.Q..V.T...LT...FSK....AM........L.........VC....K.. 
KF498977                    .......L.....NT..........T..............F............V.TTV......LS.I...AL........L.................. 
KF498978                    ........................ST...A.L........F............V.TT..P....FS.....AP...ST...................... 
KF498979                    ........................ST...A.L........F............V.TT..P....FS.....AP...ST...................... 
KF498980                    .......L......T..........T..............F............V.TT..P....FS.I...AL........................... 
KF498981                    .............................A..........F.........L..V.T.........S.I...AP........................... 
KF498982                    ........................ST...A.L........F.........I..V.TT..P....FS.....AP...ST...................... 
KF498983                    ........................ST...A.L........F............V.TT..P....FS.....AP...ST...................... 
KF498984                    .............................A..........F.........L..V.T.........S.I...A............................ 
KF498985                    .......L.....NT..........T............V.F............V.TT..P....FS.I...AL........................... 
KF498986                    .A.....L......T.......T..T...A..........F.....T......V.TT..P.L..FSKI...AP........................... 
KF498987                    ........................ST...A.L........F............V.TT..P....FS.....AP...ST...................... 
KF498988                    .............................A..........F.........L..V.T.........S.I...AS........................... 
KF498989                    ........................ST...A.L........F............V.TT..P....FS.....AP...ST...................... 
KF498990                    .............................A..........F.........L..V.T.........S.I...AP........................... 
KF498991                    .......L.....NT..........T..............F............V.TT..P....FS.I...AL........................... 
KF498992                    ........................ST...A.L........F............V.TT..P....FSK....AP...ST...................... 
KF498993                    .............................A.L........F............V.TT..P....FS.....AP...ST...................... 
KF498994                    .......L.....NT..........T...........S..F.V..........V.TT..P....FS.I...AL........................... 
KF498995                    ...................I....K....A....-------.........L..V.T.........S.....AP........................... 
KF498996                    ........................ST...A.L........F............V.TT..P....FS.....AP...ST...................... 
KF498997                    .............................A..........F.........L..V.T.........S.I...AP........................... 
KF498998                    ........................ST...A.L.....---G............V.TT..P....FS.....AP...ST...................... 
KF498999                    T.......................ST...A.L........F............V.TT..P....FS.....AP...STA..................... 
KF499000                    .......L.....NT..........T..............F............V.TT...L...FS.I...AL........................... 
KF499001                    .......L.....NT..........T..............F............V.TT..P....FS.I...AL........................... 
KF499002                    .......L.....NT..........T..............F............V.TT..P....FS.I...AL........................... 
KF499003                    ........................ST...A.L........F............V.TT..P....FS.....AP...ST...................... 
KF499004                    ........................ST...A.L........F..........H.V.TT..P....FS.....AP...ST...................... 
KF499005                    ...................I....ST...A.L......--..........I..VTTT........S.I...AP........................... 
KF499006                    .............................A..........F.........L..V...........S.....AP........................... 
KF499007                    .............................A.......S..F.........L..V.T.....AAV.S.I...APK.......................... 
KF499008                    ...................T...--T...A.LH.......F............V.TT..P....FS.....AP...ST...................... 
KF499009                    .......L.....NT..........T..............F............V.TT..P....FS.I...AL........................... 
KF499010                    ........................ST...A.L........F............V.TT..P.L..FS.....APK..ST...................... 
KF499011                    .............E..........ST...A.L.....................V.TT..P....FS.....AP...ST...................... 
KF499012                    ........................ST...A.L........F............V.TT..P....FS.....AP...ST...................... 
KF499013                    .......L......T.......T..T.............S......T......V.TT..P.L..FSKI...AP........................... 
KF499014                    .............................A..........F.........L..V.T.........S.I...AP........................... 
KF499015                    ........................ST...A.L........F............V.TT..P....FS.....AP...ST...................... 
Clustal Consensus           :.*:***::****::**** **.   ***:* .*       *.***:*    *. . .*   :: :: *.*. :*:. :**:*********  ****:** 

                                    210       220       230       240       250       260       270       280       290       300         
                            ....|....|....|....|....|....|....|....|....|....|....|....|....|....|....|....|....|....|....|....|
gi|59418|emb|X02763.1       TIPQSLDSWWTSLNFLGGSPVCLGQNSQSPTSNHSPTSCPPICPGYRWMCLRRFIIFLFILLLCLIFLLVLLDYQGMLPVCPLIPGSTTTSTGPCKTCTT 
gi|221497|dbj|D00329.1|     ..................T..........QI.S....C....................C............................S............ 
gi|59585|emb|X04615.1|      ..................A.T.P..................T.........................................L..TS.......R...I 
gi|59439|emb|X65259.1|      ..................TT.....................T.............................................S.......R.... 
gi|452617|emb|X75657.1|     ..................A.............S......................................................S.......R.... 
gi|59422|emb|X69798.1|      ..................L.G.P...........L......T.........................................L................ 
gi|6983934|gb|AF160501.1|   ..................V...P.L...........I....T.............................................S............ 
gi|22135696|gb|AY090454.1|  .................VP.G.P.......I...L......T.........................................L................ 
KF498977                    ..................TT.....................T.............................................S.......R.... 
KF498978                    ..................A.T.P..KF........................................................L..TS....RA.....I 
KF498979                    ..................A.T.P...L........................................................L..TS...........I 
KF498980                    .............K....TT.....................T.............................................S...V...R.... 
KF498981                    .................................................................................................... 
KF498982                    ..................A.T.P...L........................................................L..TS...........I 
KF498983                    ..................A.T.P...L........................................................L..TS...........I 
KF498984                    .................................................................................................... 
KF498985                    ..................TT.....................T.............................................S...V...R.... 
KF498986                    ..................TT.....................A.............................................S.......R..M. 
KF498987                    ..................A.T.P...L........................................................L..TS...........I 
KF498988                    .................................................................................................... 
KF498989                    ..................A.T.P...L........................................................L..TS...........I 
KF498990                    .................................................................................................... 
KF498991                    ..................NT.....................T.............................................S...V...R.... 
KF498992                    ..................A.T.P...L........................................................L..TS...........I 
KF498993                    ...R..............A.K.P...L........................................................L..TS...........I 
KF498994                    ..................TT.....................T.............................................S...V...R.... 
KF498995                    .........................................T.............................................S...V...R.... 
KF498996                    ..................A.T.P...L........................................................L..TS...........I 
KF498997                    .................................................................................................... 
KF498998                    ..................A.T.P...L........................................................L..TS..I........I 
KF498999                    ..................A.T.P...L........................................................L..TS...........I 
KF499000                    ..................TT.....................T.............................................S.......R.... 
KF499001                    ..................TT.....................T.............................................S...V...R.... 
KF499002                    ..................TT.....................T.............................................S...V...R.... 
KF499003                    ..................A.T.P...L........................................................L..TS...........I 
KF499004                    ..................A.T.P...L........................................................L..TS...........I 
KF499005                    ...................................................................................L..TS...........I 
KF499006                    .................................................................................................... 
KF499007                    ..................A...R............................................................................. 
KF499008                    ..................A.T.P...L........................................S......R........L..TS...........I 
KF499009                    ..................TT.....................T.............................................S...V...R.... 
KF499010                    ..................A.A.P...................S........................................L..TS...........I 
KF499011                    ..................A.T.P...L........................................................L..TS...........I 
KF499012                    ..................A.T.P...L.................................................LP-....L..TS............ 
KF499013                    ..................TT.....................T.............................................S.......R..M. 
KF499014                    .................................................................................................... 
KF499015                    ..................A.T.P...L........................................S......R........L..TS...........I 
Clustal Consensus           ***:*********:***  . * * : **  *.* * .*** .*************** ******** ******:*:  ****:**::** . .*:**   

                                    310       320       330       340       350       360       370       380       390       400         
                            ....|....|....|....|....|....|....|....|....|....|....|....|....|....|....|....|....|....|....|....|
gi|59418|emb|X02763.1       PAQGNSMFPSCCCTKPTDGNCTCIPIPSSWAFAKYLWEWASVRFSWLSLLVPFVQWFVGLSPTVWLSAIWMMWYWGPSLYSIVSPFIPLLPIFFCLWVYI 
gi|221497|dbj|D00329.1|     ....T...........M..................................................V............N.L...M............. 
gi|59585|emb|X04615.1|      ....T...........S................RF.............................................N.L...L............. 
gi|59439|emb|X65259.1|      ....T..Y........S...............G.F......A.........................V..............L...L............. 
gi|452617|emb|X75657.1|     L...T........S..S...............G.F......A...............A.........V............N.L................. 
gi|59422|emb|X69798.1|      L...T........S..S..............LG........A.........Q....C.........LV...I.....N.C..L..........CY...S. 
gi|6983934|gb|AF160501.1|   .......Y........S............................................................N..N.L................. 
gi|22135696|gb|AY090454.1|  L...T...........S...............G........A.........Q....C.........LV...I.....N.C..L..........CY..AS. 
KF498977                    ....T..Y........S.........A.....G.F....------------------------------------------------------------- 
KF498978                    ....T...........S................RF....------------------------------------------------------------- 
KF498979                    ....T...........S................RF....------------------------------------------------------------- 
KF498980                    TV..T..Y........S...............G.F...-------------------------------------------------------------- 
KF498981                    ..................................F....------------------------------------------------------------- 
KF498982                    ....T...........S................RF......................------------------------------------------- 
KF498983                    ....T...........S................RF...-------------------------------------------------------------- 
KF498984                    ...................................----------------------------------------------------------------- 
KF498985                    TV..T..Y........S...........A...G.F....------------------------------------------------------------- 
KF498986                    T...T..Y........S.R.....L.......G.F.---------------------------------------------------------------- 
KF498987                    ....T...........S.....W..........RF......................------------------------------------------- 
KF498988                    .........................................................------------------------------------------- 
KF498989                    ....T...........S................RF......................------------------------------------------- 
KF498990                    .........................................................------------------------------------------- 
KF498991                    TV..T..Y........S...............G.F......A...............------------------------------------------- 
KF498992                    ....T...........S............S...RF.R.-------------------------------------------------------------- 
KF498993                    ....T...........S..................----------------------------------------------------------------- 
KF498994                    TV..T..Y........S...............G.F......A...............D------------------------------------------ 
KF498995                    TV..T..Y........S...............G.F......A...............D------------------------------------------ 
KF498996                    ....T...........S............S...RF----------------------------------------------------------------- 
KF498997                    .............................S.......--------------------------------------------------------------- 
KF498998                    ....T...........S................RF......I...............------------------------------------------- 
KF498999                    ....T...........S................RF......................------------------------------------------- 
KF499000                    ....T..Y........S...............G.F......A...............------------------------------------------- 
KF499001                    TV..T..Y........S...............G.F......A...............------------------------------------------- 
KF499002                    TV..T..Y........S............S..G...P--------------------------------------------------------------- 
KF499003                    ....T...........S................RF....------------------------------------------------------------- 
KF499004                    ....T...........S.................F....------------------------------------------------------------- 
KF499005                    ....T...........S................RF......................------------------------------------------- 
KF499006                    ................................G......------------------------------------------------------------- 
KF499007                    .....................................--------------------------------------------------------------- 
KF499008                    ....T...........S................RF......................------------------------------------------- 
KF499009                    TV..T..Y........S...............G.F....------------------------------------------------------------- 
KF499010                    ....TY......S........................--------------------------------------------------------------- 
KF499011                    ....T...........S................R..---------------------------------------------------------------- 
KF499012                    ....T...........S................R.----------------------------------------------------------------- 
KF499013                    T...T..Y........S...............G.F.---------------------------------------------------------------- 
KF499014                    .........................................................------------------------------------------- 
KF499015                    ....T...........S................RF......................------------------------------------------- 
Clustal Consensus            .**. *:****.:** * *** * *.*: *:.::                                                                  
